# Alkaloid-driven multi-target synergy of *Tripterygium wilfordii* polyglycosides overcomes cisplatin resistance in ovarian cancer by coordinated inhibition of PTPN11/EGFR/JAK signaling

**DOI:** 10.3389/fphar.2025.1686526

**Published:** 2025-10-17

**Authors:** Bing Lin, Minxin Zhang, Ying Wang

**Affiliations:** ^1^ Department of Pharmacy, Fuzong Clinical Medical College of Fujian Medical University, Fuzhou, China; ^2^ Department of Pharmacy, 900th Hospital of PLA Joint Logistic Support Force, Fuzhou, China

**Keywords:** Tripterygium wilfordii polyglycoside, ovarian cancer, cisplatin resistance, network pharmacology, multi-target therapy, alkaloids

## Abstract

**Objective:**

*Tripterygium wilfordii* polyglycoside (TWP) is a standardized extract from *T. wilfordii* Hook. f. and an oral prescription drug approved by the China Food and Drug Administration (now NMPA) for clinical use in inflammatory and autoimmune diseases. Leveraging its existing clinical approval, elucidating its anti-tumor mechanisms has high translational value for expanding its indications into oncology. This study aimed to clarify whether TWP can overcome cisplatin resistance in ovarian cancer and to explore a mechanism potentially centered on its alkaloid constituents through an integrated “prediction–validation” strategy.

**Methods:**

UPLC-QTOF-MS was used for chemical profiling. Network pharmacology predicted putative targets, validated by GEO transcriptomic datasets. Key alkaloid–target interactions were examined by molecular docking and 100-ns MD simulations. *In vitro* assays (CCK-8, Annexin V-FITC/PI, Western blot) in cisplatin-resistant A2780/DDP cells confirmed phenotypic and mechanistic effects.

**Results:**

Thirty-eight constituents were identified, including 18 alkaloids. Five core targets (EGFR, JAK1, JAK2, PTPN11, SRD5A1) were pinpointed by network–clinical integration. Several alkaloids ranked among the top compounds by network degree, exhibited strong predicted binding affinities (ΔG ≤ −7 kcal/mol), and formed stable complexes in molecular dynamics simulations. Functionally, TWP reduced viability, induced apoptosis, and de-phosphorylated EGFR, JAK1/2, and PTPN11, downregulated SRD5A1, and suppressed PI3K-AKT, JAK-STAT, and ERK-MAPK signaling.

**Conclusion:**

Our findings suggest that alkaloids in TWP may exert multi-target synergy to disrupt key survival pathways driving cisplatin resistance in ovarian cancer. These mechanistic insights not only rationalize its observed anti-tumor activity but also support its potential clinical repurposing from an approved anti-inflammatory drug to an oncology therapeutic.

## Graphical Abstract

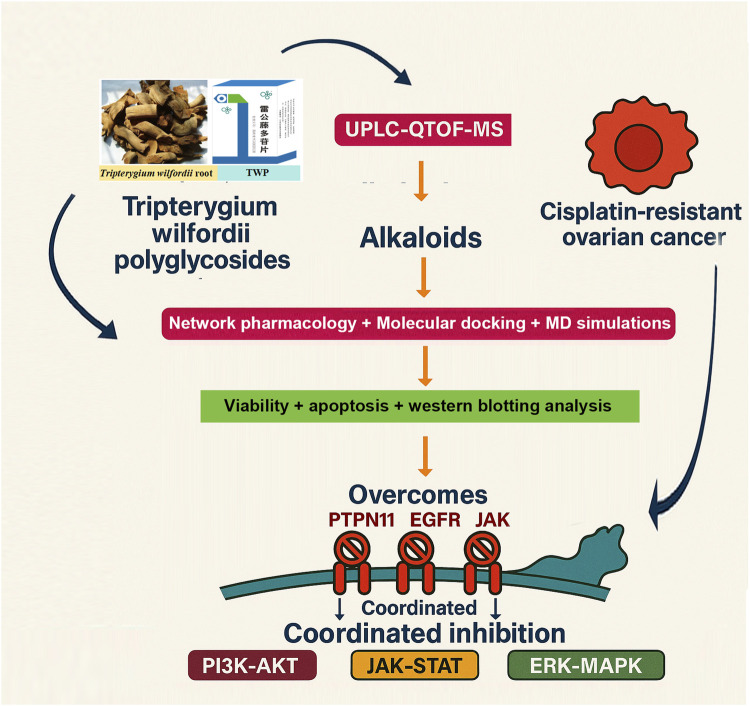

## 1 Introduction

Ovarian cancer remains a formidable challenge in gynecological oncology, characterized by insidious progression, late-stage diagnosis, and high mortality rates. Despite initial responsiveness to cytoreductive surgery and platinum-based chemotherapy, approximately 70% of patients with advanced disease experience recurrence within 18 months, subsequently developing chemoresistance that severely compromises therapeutic efficacy and survival outcomes (Murphy et al., 2021). This escalating crisis of platinum resistance underscores an urgent unmet need for novel therapeutic strategies capable of overcoming treatment-refractory disease.

In this context, Traditional Chinese Medicine (TCM) has garnered significant attention for its holistic, multi-component, and multi-target therapeutic philosophy. Accumulating evidence has validated the efficacy of various TCM-derived agents against ovarian cancer. For instance, compounds such as berberine and curcumin have been shown to suppress tumor progression and induce apoptosis by modulating critical oncogenic pathways like PI3K/Akt and EGFR, with notable efficacy even in cisplatin-resistant cells (Zhang et al., 2025; Liu et al., 2023). Furthermore, other agents like ginsenoside Rg6 specifically target chemoresistance, enhancing cisplatin sensitivity through mechanisms such as the induction of autophagy (Xue et al., 2025). These examples highlight the capacity of TCM-derived compounds to combat ovarian cancer through diverse and synergistic mechanisms.

Among TCM-derived agents, *Tripterygium wilfordii* polyglycoside (TWP) is a standardized oral extract from *T. wilfordii* Hook. f. and an approved prescription drug by the China Food and Drug Administration (now NMPA) for clinical use in inflammatory and autoimmune diseases. Previous studies have demonstrated that TWP possesses significant anti-tumor effects, including the ability to inhibit ovarian cancer cell proliferation, suppress migration and invasion, and reverse cisplatin resistance in drug-resistant cell lines (Zhan et al., 2023; Ma et al., 2023). Nevertheless, the precise molecular mechanisms underlying these multi-faceted effects remain incompletely elucidated. Notably, while the anti-tumor activity of TWP is often attributed to its well-documented diterpenoid constituents, the specific contribution of its large family of alkaloid compounds—a class known for potent bioactivity as exemplified by agents like berberine—remains a critical and under explored area, impeding the rational optimization of TWP-based regimens.

To bridge this critical knowledge gap, we employed a multi-dimensional integrative approach combining UPLC/Q-TOF-MS phytochemical profiling, network pharmacology, microarray-based transcriptomic analysis, and advanced molecular modeling techniques. This strategy enabled systematic deconvolution of TWP’s chemical composition, identification of its bioactive constituents, prediction of therapeutic targets, and validation of their clinical relevance in ovarian cancer pathogenesis. Based on our initial objective to investigate the under explored components of TWP, our findings compellingly demonstrate that alkaloids, far from being secondary players, constitute a primary class of active components driving TWP’s anti-ovarian cancer effects. This was evidenced by their superior efficacy in targeting key chemoresistance pathways in our network pharmacology models. Through integrated network analysis and clinical transcriptomic data, we identified five core targets (EGFR, JAK1, JAK2, PTPN11, and SRD5A1) that are differentially expressed in ovarian cancer tissues and serve as central nodes in TWP’s mechanism of action. Subsequent molecular docking and dynamics simulations confirmed stable, high-affinity binding between TWP alkaloids and these targets, providing structural insights into their interactions. Crucially, experimental validation demonstrated that TWP simultaneously modulates these targets to coordinately suppress PI3K-AKT, JAK-STAT, and ERK-MAPK signaling pathways, thereby inducing apoptosis and overcoming cisplatin resistance in ovarian cancer cells.

This study elucidates a novel mechanistic framework by which TWP’s alkaloid constituents exhibit multi-target synergistic effects against platinum-resistant ovarian cancer. By deliberately shifting the scientific focus from the well-trodden path of diterpenoids, our findings establish that TWP’s alkaloids play a pivotal, not merely a supplementary, role in modulating key molecular targets (EGFR, JAK1/2, PTPN11, SRD5A1) and associated signaling pathways (PI3K-AKT, JAK-STAT, and ERK-MAPK). These results complement existing knowledge of TWP’s anti-tumor properties and provide new insights for developing more effective therapeutic strategies against chemoresistant ovarian cancer.

## 2 Materials and methods

### 2.1 Chemicals and reagents

Chromatographic-grade methanol and acetonitrile were obtained from Merck and Sigma-Aldrich (Darmstadt, Germany). Deionized water was prepared using a Milli-Q system (Millipore, Bedford, MA, United States). Tripterygium wilfordii polyglycoside (TWP) was provided by Jiangsu Meitong Pharmaceutical Co., Ltd. (Jiangsu, China). RIPA lysis buffer was from Beyotime (Shanghai, China). Primary antibodies: SRD5A1 (ab167606, 1:2000, rabbit; Abcam, Waltham, MA, United States); JAK1 (PT0658R, 1:1000, rabbit), JAK2 (PT0503R, 1:2000, rabbit), Bax (PT0301R, 1:2000, rabbit), Bcl-2 (PT0487R, 1:2000, rabbit), phospho-STAT3 (Tyr705; YP0251, 1:2000, rabbit), STAT3 (PT0911R, 1:2000, rabbit), and Akt (PT0654R, 1:2000, mouse) from ImmunoWay (Jiangsu, China); cleaved caspase-3 (#96641, 1:1000, rabbit), EGFR (#4267, 1:1000, rabbit), phospho-EGFR (#3777, 1:1000, rabbit), PTPN11/SHP2 (#3397, 1:2000, rabbit), phospho-PTPN11 (#5431, 1:1000, rabbit), phospho-ERK1/2 (#4370, 1:2000, rabbit), ERK1/2 (#4695, 1:2000, rabbit) from Cell Signaling Technology (Danvers, MA, United States); phospho-Akt (AA329, 1:2000, rabbit), phospho-JAK1 (AF5857, 1:2000, rabbit), phospho-JAK2 (AF1486, 1:2000, rabbit), and GAPDH (AF0006, 1:10000, mouse) from Beyotime (Shanghai, China).

### 2.2 UPLC/Q-TOF-MS profiling

#### 2.2.1 Sample preparation

TWP (20.0 mg) was weighed into a 10 mL volumetric flask, dissolved by sonication in methanol, brought to volume, vortex-mixed, and filtered (0.22 µm).

#### 2.2.2 LC–MS conditions

Chromatography used an Agilent 1290 UPLC with a Waters ACQUITY UPLC XBridge BEH C18 column (2.1 × 50 mm, 1.7 μm). Mobile phase: acetonitrile (A) and water with 0.1% formic acid (B), 0.3 mL/min, 35 °C. Gradient: 0–10 min, 20% A; 10–60 min, 20%–45% A; 60–90 min, 45% A; 90–120 min, 45%–85% A; 120–150 min, 85% A; 150–170 min, 85%–90% A. Injection: 5 μL.

An Agilent 6520 Q-TOF with ESI in positive mode acquired mass spectra (source 350 °C; drying gas 10 L/min; nebulizer 40 psi; fragmentor 130 V; capillary 3500 V; 1 spectrum/s, 2 GHz dynamic range; m/z 50–1200). MS/MS used the same source settings.

#### 2.2.3 Compound identification

A Tripterygium database was compiled from CNKI, Wanfang, PubMed, Web of Science, SciFinder, and Reaxys. Constituents were annotated by accurate mass, MS/MS fragmentation, and retention time, and confirmed against reference standards when available. Molecular formulae were inferred from exact mass and isotopic patterns. Compounds were classified as alkaloids, diterpenoids, triterpenoids, or other.

### 2.3 Target prediction and disease target collection

Active constituents were selected from the 38 identified compounds based on the criteria of Oral Bioavailability (OB) ≥ 30% and Drug-Likeness (DL) ≥ 0.18, yielding 26 compounds for target prediction. Human targets were predicted using SwissTargetPrediction (probability ≥0.7), excluding low-confidence predictions. Ovarian cancer-associated genes were retrieved from GeneCards, OMIM, and CTD using the keywords “ovarian neoplasm,” “ovarian tumor,” and “ovarian carcinoma.” Duplicates and non-human entries were removed. Overlap with TWP-predicted targets was computed in R (v4.2.0, VennDiagram); UniProt IDs were used downstream.

### 2.4 Network construction and analysis

A compound–target bipartite network was built in Cytoscape 3.9.0. Topological parameters (degree, betweenness, closeness) prioritized key constituents and targets. Overlapping targets were submitted to STRING v11.5 (*Homo sapiens*; minimum interaction score ≥0.4) including experimental, curated database, and text-mining evidence (neighbourhood, gene fusion, and co-occurrence excluded). Networks were visualized in Cytoscape. CytoHubba identified hub genes using Maximal Clique Centrality (MCC), and MCODE detected densely connected subnetworks using standard parameters (degree cutoff = 2, node score cutoff = 0.2, k-core = 2, max depth = 100).

### 2.5 Functional enrichment

GO and KEGG enrichment used DAVID 6.8 (*H. sapiens* background), with p < 0.01 and Benjamini–Hochberg correction. GO terms were categorized into biological process (BP), cellular component (CC), and molecular function (MF). The top 10 terms in each category were visualized as bubble plots (ggplot2, enrichplot), with bubble size denoting gene count and color indicating −log10 (adjusted p).

### 2.6 Molecular docking and MD simulations

The ten highest-ranked constituents by network centrality were docked to five core targets (EGFR, JAK1, JAK2, PTPN11, SRD5A1). Ligand 3D structures were retrieved from PubChem (SDF), protonated at pH 7.4, and minimized with MMFF94 (Open Babel 3.1). Protein structures: EGFR (PDB: 1M17); JAK1 (PDB: 4E5W) and JAK2 (PDB:3IO7) kinase domains (validated high-resolution human structures with co-crystallized inhibitors); PTPN11 (PDB: 3MOW); SRD5A1 (PDB: 7C83) modeled using the AlphaFold structure (UniProt Q13675; AF-Q13675-F1) due to the lack of a suitable experimental structure. Proteins were prepared in AutoDockTools 1.5.6 (removal of waters/non-native ligands, addition of polar hydrogens, Gasteiger charges). Grid boxes were centered on catalytic pockets. Docking used AutoDock Vina 1.2.2 (exhaustiveness = 8; 20 runs/ligand). Lowest-energy poses (ΔGbind) were retained; binding modes were visualized in PyMOL 3.0 and annotated with LigPlot+ 2.2.

For MD, 100-ns all-atom simulations were performed in GROMACS 2024.3. Proteins used Amber99SB-ILDN; ligands were parameterized with GAFF via ACPYPE/Antechamber (AM1-BCC charges). Complexes were solvated in a dodecahedral box of SPC216 water with 0.15 M NaCl. Energy minimization employed steepest descent (5,000 steps) to max force <1000 kJ/mol/nm. Equilibration: 500 ps NVT at 300 K (heavy-atom restraints) and 1,000 ps NPT at 300 K with gradually reduced restraints. Production: 300 K, 1 bar, v-rescale thermostat (τT = 0.1 ps), Parrinello–Rahman barostat (τP = 2.0 ps). PME handled electrostatics (real-space cutoff 1.0 nm); van der Waals used a force-switch at 0.9–1.0 nm. Bonds to hydrogens were constrained with LINCS (2 fs time step). Trajectories were saved every 10 ps? RMSD and RMSF were computed with GROMACS tools and plotted in R.

### 2.7 Microarray analysis and experimental validation

#### 2.7.1 Clinical transcriptomics

Four GEO datasets (GSE18520, GSE26712, GSE27651, GSE54388) were analyzed. Preprocessing and differential expression used limma (background correction, normalization, probe summarization). DEGs were defined as adjusted p < 0.05 and |log2 fold change| ≥ 1. DEGs were cross-referenced with hub genes to derive core targets.

#### 2.7.2 Cell culture and treatments

Human ovarian cancer SKOV3 and cisplatin-resistant A2780/DDP cells (Changsha Abiowell Biotechnology Co., Ltd., Changsha, China) were cultured in DMEM with 10% FBS and 1% penicillin/streptomycin at 37 °C in 5% CO_2_. TWP stocks were prepared in DMSO; final DMSO ≤0.1%. Cisplatin was used at 10 μM where indicated.

#### 2.7.3 Cell viability

CCK-8 (Beyotime, Shanghai, China) assessed viability. Cells were seeded in 96-well plates (5 × 10^3^ cells/well), incubated overnight, and treated with cisplatin (10 μM) and TWP (0, 5, 10, 20 μg/mL) for 24 h. CCK-8 reagent (10 μL) was added for 2 h at 37 °C; absorbance at 450 nm was read (BioTek Instruments).

#### 2.7.4 Apoptosis

Annexin V-FITC/PI staining (Elabscience) followed the manufacturer’s protocol. After 24 h TWP (0, 5, 10, 20 μg/mL), cells were harvested, washed, stained for 15 min at room temperature in the dark, and analyzed on a CytoFLEX nano flow cytometer (Beckman Coulter).

#### 2.7.5 Western blot

Total protein was extracted with RIPA plus protease/phosphatase inhibitors (Wuhan Cobio, Wuhan, China) and quantified by BCA (Wuhan Cobio). Equal protein (30 μg) was separated by 10% SDS-PAGE, transferred to PVDF (Millipore), blocked with 5% non-fat milk (1 h, room temperature), and incubated overnight at 4 °C with primary antibodies against EGFR, phospho-EGFR, JAK1, phospho-JAK1, JAK2, phospho-JAK2, PTPN11, phospho-PTPN11, SRD5A1, Akt, phospho-Akt, STAT3, phospho-STAT3, ERK1/2, phospho-ERK1/2, Bcl-2, Bax, cleaved caspase-3, and GAPDH. HRP-conjugated secondary antibodies (1:5000) and ECL (GE Healthcare) were used for detection. Bands were quantified with ImageJ v1.53t and normalized to GAPDH.

### 2.8 Statistical analysis

Unless otherwise indicated, data represent mean ± SD from n = 3 independent experiments (each with ≥3 technical replicates). One-way ANOVA with Tukey’s *post hoc* test was used for multiple comparisons (GraphPad Prism 8.0). Two-sided p < 0.05 was considered statistically significant.

## 3 Results

### 3.1 UPLC/Q-TOF-MS-based qualitative profiling of TWP

**FIGURE 1:**
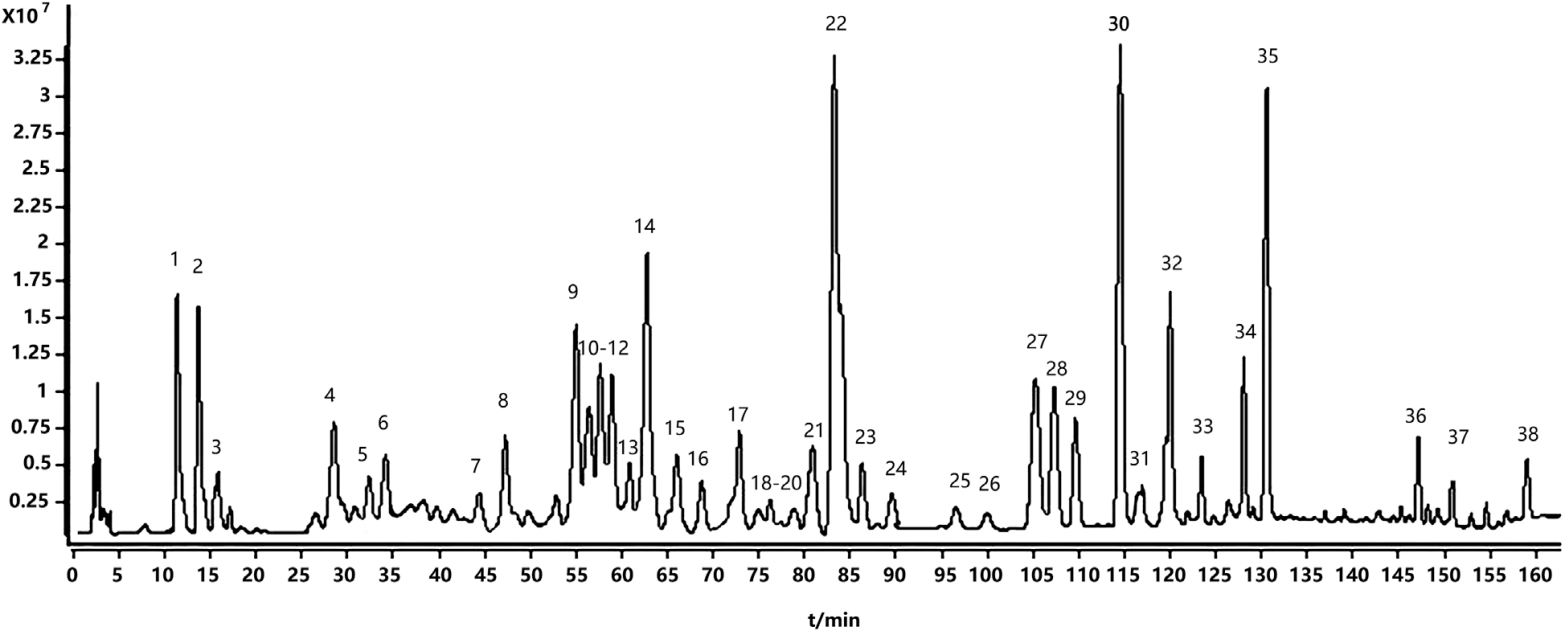
The total ion chromatogram of TWP in positive ion mode by UPLC-QTOF-MS.

UPLC/Q-TOF-MS in positive and negative ion modes identified 38 constituents; positive mode yielded superior signal and was used for detailed analysis (Figure 1). Based on [M + H]^+^ ions, fragmentation patterns, standards, and literature, we annotated 18 alkaloids, 8 diterpenoids, 8 triterpenoids, and 4 others (Table 1). The fact that alkaloids represent the most numerous chemical class identified (18 of 38 total compounds) prompted us to hypothesize their significant contribution to TWP’s overall bioactivity. This hypothesis was subsequently tested and strongly supported by our network pharmacology analysis (see Section 3.3), which revealed that alkaloid constituents were the most highly connected nodes in the compound-target network, suggesting they are the primary mediators of TWP’s effects on ovarian cancer targets.

**TABLE 1:** UPLC-QTOF-MS identification of 38 chemical constituents in TWP.

Peak no.	Compound name	Molecular formula	[M + H]^+^/[M + Na]^+^ (*m/z*)	RT/min	DL
1	Triptofordinine A2	C_41_H_43_NO_12_	742.7796	11.25	0.47
2	Wilforjine	C_36_H_45_NO_17_	764.2841	14.08	0.72
3	Hypodiol	C_30_H_50_O_2_	443.1555	15.78	0.18
4	Wilfordinine B	C_38_H_47_NO_19_	822.2888	28.85	0.74
5	Neoeuonymine	C_36_H_45_NO_17_	764.2824	32.55	0.54
6	Tripterifordin	C_20_H_30_O_3_	319.2307	34.44	0.49
7	Wilfordinine A	C_36_H_45_NO_17_	764.2828	44.42	0.88
8	Euonine	C_38_H_47_NO_18_	806.2956	47.12	0.2
9	Triptonodiol	C_21_H_30_O_4_	347.2259	49.95	0.42
10	Wilfortrine	C_41_H_47_NO_20_	874.2866	51.15	0.15
11	Tripterygiol	C_22_H_28_O_8_	421.4529/443.1688	57.52	0.16
12	Hypoglaunine A	C_41_H_47_NO_20_	874.2832	59.05	0.78
13	Euonine	C_38_H_47_NO_18_	806.295	60.75	0.2
14	Triptoquinone B	C_20_H_26_O_4_	331.1936	62.85	0.32
15	2α, 3α, 23-trihydroxyursane-12-en-28 oic acid	C_30_H_48_O_5_	489.3245	65.93	0.71
16	Wilfordine	C_43_H_49_NO_19_	884.304	68.91	1.05
17	9′-O-Acetylwilfortrine	C_43_H_49_NO_21_	916.2919	73.02	0.56
18	Hypoglaunine D	C_41_H_47_NO_19_	858.2891	75.05	0.94
19	Aurantiamide acetate	C_27_H_28_N_2_O_4_	445.1561	76.14	0.52
20	Euojaponine D	C_41_H_47_NO_17_	826.2944	79.26	0.7
21	Wilfornine A	C_45_H_51_NO_20_	926.3116	80.78	0.4
22	Wilfordinine F	C_43_H_49_NO_18_	868.308	83.81	1.1
23	2β,22β-Dihydroxy-3,21-dioxo-24-carboxyl-29-nor-friedelan methyl ester	C_30_H_46_O_6_	503.3372	86.27	0.26
24	6α-hydroxytriptocalline A	C_28_H_44_O_5_	461.1522	89.95	−0.1
25	Mayteine	C_43_H_49_NO_18_	868.3025/890.2850	96.35	0.61
26	9′-O-furanoylwilfordine	C_48_H_51_NO_21_	978.3053/1000.2856	99.92	0.53
27	Orthosphenic acid	C_30_H_48_O_5_	489.3596	105.13	−0.09
28	Kaempferol	C_15_H_18_O_5_	279.1602	107.18	0.24
29	Celastrol	C_29_H_38_O_4_	451.2854/473.3638	109.83	0.63
30	Salazinic acid	C_19_H_14_O_10_	403.2347/425.2162	114.88	0.76
31	Nobiletin	C_21_H_22_O_8_	403.2338/425.2157	116.78	0.52
32	Triptobenzene C	C_20_H_26_O_4_	331.2846/353.2676	120.08	0.94
33	Zhebeiresinol	C_14_H_16_O_6_	281.248	123.55	0.19
34	Triptonoterpene	C_20_H_28_O_2_	301.2114	128.28	0.28
35	Demethylregelin	C_30_H_46_O_4_	471.3468	130.45	1.14
36	Salaspermic acid	C_30_H_48_O_4_	473.3486	147.02	0.63
37	Triptoditerpenic acid B	C_20_H_24_O_4_	329.243	150.79	0.36
38	Triptophenolide	C_20_H_24_O_3_	313.3271	159.08	0.44

### 3.2 Screening for ovarian cancer-related targets of TWP

**FIGURE 2:**
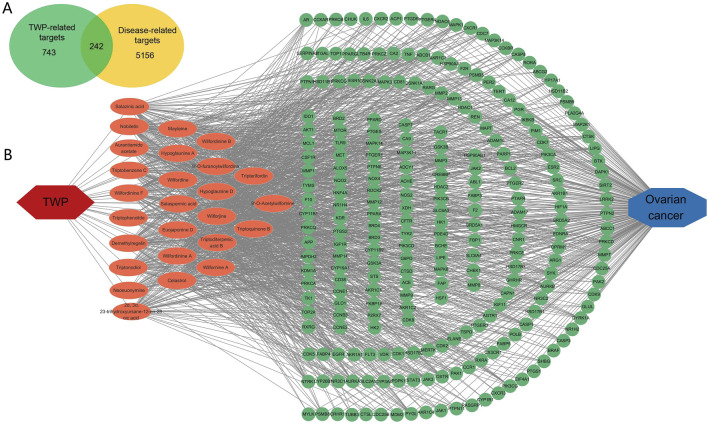
Network analysis of TWP and ovarian cancer targets. **(A)** Venn diagram illustrating overlap between TWP-predicted targets (n = 743) and ovarian cancer-related genes (n = 5,156), highlighting 242 shared targets. **(B)** Compound-target network: Yellow nodes represent TWP compounds; blue nodes represent targets; edges indicate interactions.

Potential molecular targets of TWP constituents were predicted using SwissTargetPrediction, yielding 743 unique targets. Ovarian cancer-associated genes (n = 5,156) were curated from GeneCards, OMIM, and CTD databases. Venn diagram analysis identified 242 overlapping targets between TWP-predicted targets and ovarian cancer-related genes (Figure 2A), suggesting these may mediate TWP’s therapeutic effects against ovarian cancer.

### 3.3 Network analysis of TWP and ovarian cancer targets

A compound-target network was constructed to visualize interactions between TWP constituents and their predicted targets. The bipartite network (Figure 2B) comprised 268 nodes (26 compounds, 242 targets) and 1,274 edges, reflecting TWP’s multi-component, multi-target pharmacology. Topological analysis identified highly connected compounds, likely representing key pharmacologically active constituents. The top 10 compounds by degree (Table 2) were predominantly alkaloids (e.g., wilfordinine A, wilforidine, wilfornine A, wilfortrine, euojaponine D), consistent with chemical profiling and suggesting alkaloids as primary mediators of TWP’s anti-ovarian cancer effects.

**TABLE 2:** Top 10 TWP constituents ranked by network degree.

No.	Compound	Class	Degree	Betweenness centrality
1	Wilfordinine A	Alkaloids	68	0.0812
2	Hypoglaunine D	Alkaloids	66	0.0824
3	Wilfordinine F	Alkaloids	66	0.0520
4	Mayteine	Alkaloids	66	0.0553
5	Euojaponine D	Alkaloids	65	0.0676
6	Wilfornine A	Alkaloids	64	0.0445
7	Demethylregelin	Triterpenoids	63	0.0838
8	9′-O-furanoylwilfordine	Alkaloids	59	0.0426
9	Nobiletin	Flavonoid	58	0.0958
10	Triptoquinone B	Diterpenoids	56	0.0874

The 26 TWP, constituents selected for network pharmacology analysis are categorized into four classes: 13 alkaloids, 6 diterpenoids, 4 triterpenoids, and 3 other compounds. For topological feature comparison, the average degree of alkaloids is 57.3, while that of non-alkaloid compounds (including diterpenoids, triterpenoids, and others) is 31.8. The significantly higher average degree of alkaloids indicates their stronger ability to interact with ovarian cancer-related targets, further supporting their central role in mediating TWP’s multi-target therapeutic effects against cisplatin-resistant ovarian cancer.

### 3.4 PPI network analysis

**FIGURE 3:**
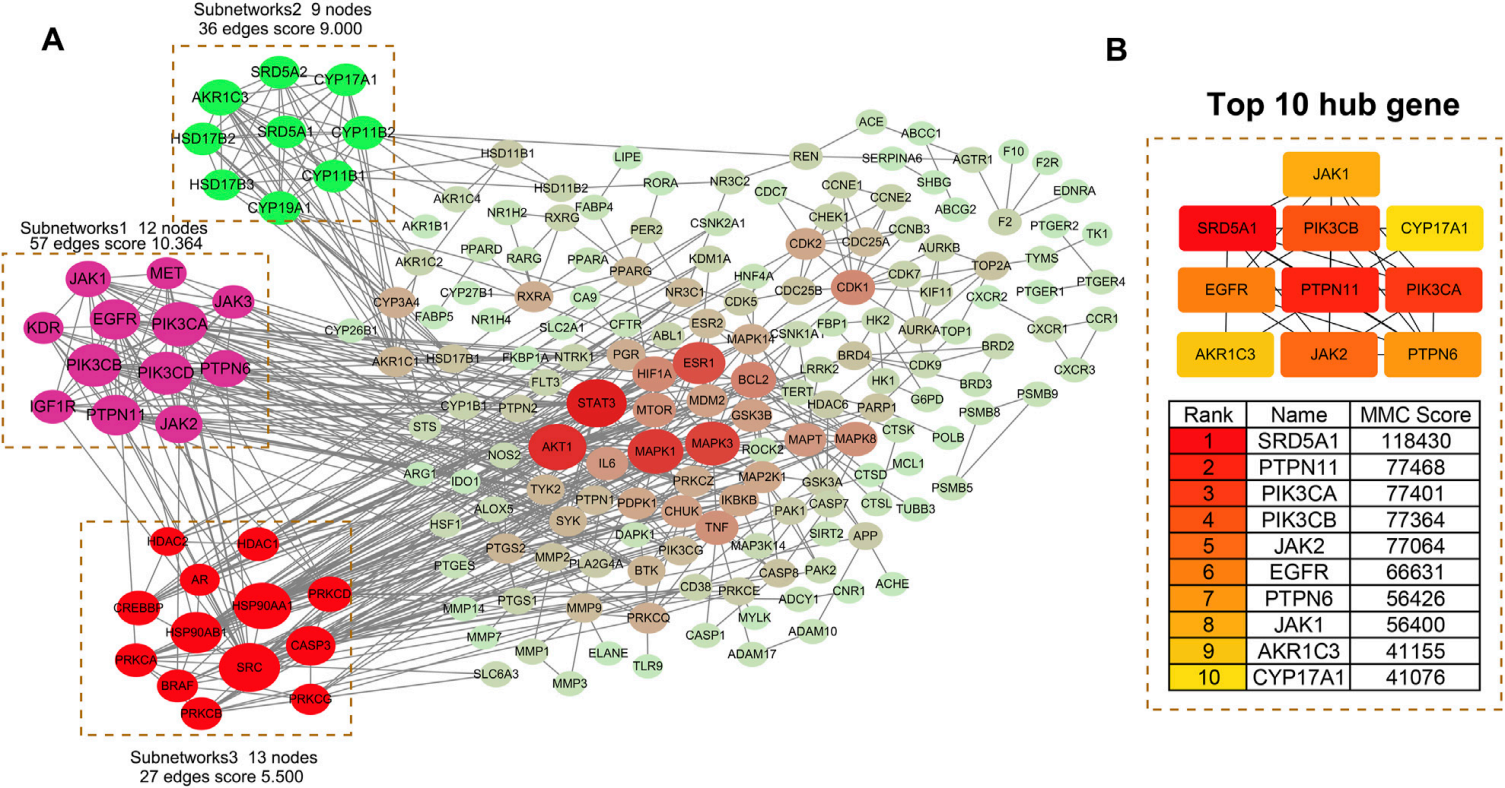
PPI network analysis of TWP targets. **(A)** Global PPI network with MCODE-identified subnetworks: Subnetwork 1: Signaling pathways (score = 10.364). Subnetwork 2: Steroid hormone biosynthesis (score = 9.000). Subnetwork 3: Inflammation/apoptosis (score = 5.500). Node size reflects connectivity; edges indicate interactions. **(B)** Top 10 hub genes ranked by degree centrality (interconnected subnetwork).

A PPI network of the 242 overlapping targets was constructed using the STRING database (Figure 3A). To identify critical functional modules, the network was analyzed using both CytoHubba and MCODE plugins. MCODE identified three densely connected subnetworks: Subnetwork 1 (Score: 10.364): Enriched in signaling pathways (12 nodes, 57 edges), featuring JAK1, JAK2, EGFR, PIK3CA, PIK3CB, and PTPN11. Subnetwork 2 (Score: 9.000): Associated with steroid hormone biosynthesis (9 nodes, 36 edges), including SRD5A1, CYP17A1, and AKR1C3. Subnetwork 3 (Score: 5.500): Involved in inflammation/apoptosis regulation (13 nodes, 27 edges), containing PRKCA, PRKCB, HSP90AA1, and SRC. Concurrently, CytoHubba identified the top 10 hub genes by degree centrality (Figure 3B): SRD5A1, PTPN11, PIK3CA, PIK3CB, JAK2, EGFR, PTPN6, JAK1, AKR1C3, and CYP17A1. These hub genes were integral to the MCODE subnetworks (e.g., SRD5A1 and CYP17A1 in Subnetwork 2; JAK2 and EGFR in Subnetwork 1). Table 3 details their functions, targeting compounds, and pathways. Notably, these genes converge on cancer-related pathways (JAK-STAT, PI3K-Akt, tyrosine kinase signaling), underscoring their role in TWP’s anti-ovarian cancer effects.

**TABLE 3:** Top 10 hub genes in the PPI network: functional annotations, targeting compounds, and pathway associations.

Rank	Gene name	MMC score	Targeting compounds	Associated pathways
1	SRD5A1	41076	12	Steroid hormone biosynthesis, Ovarian steroidogenesis, Metabolic pathways
2	PTPN11	77468	11	Phospholipase D signaling pathway, Ras signaling pathway, EGFR tyrosine kinase inhibitor resistance
3	PIK3CA	77401	10	FoxO signaling pathway, Phospholipase D signaling pathway, Pancreatic cancer, Endometrial cancer
4	PIK3CB	77364	11	Phospholipase D signaling pathway, Pancreatic cancer, Chemokine signaling pathway
5	JAK2	77064	8	JAK-STAT signaling pathway, Prolactin signaling pathway, Necroptosis, Pathways in cancer
6	EGFR	66631	12	ErbB signaling pathway, Proteoglycans in cancer, EGFR tyrosine kinase inhibitor resistance, Bladder cancer
7	PTPN6	56426	6	B cell receptor signaling pathway, Natural killer cell mediated cytotoxicity, PD-L1 expression and PD-1 checkpoint pathway in cancer
8	JAK1	56400	5	JAK-STAT signaling pathway, Necroptosis, Measles, Th17 cell differentiation
9	AKR1C3	41155	6	Steroid hormone biosynthesis, Ovarian steroidogenesis, Metabolic pathways
10	CYP17A1	41066	4	Steroid hormone biosynthesis, Ovarian steroidogenesis, Metabolic pathways, Cortisol synthesis and secretion

### 3.5 GO enrichment and KEGG pathway analysis

**FIGURE 4:**
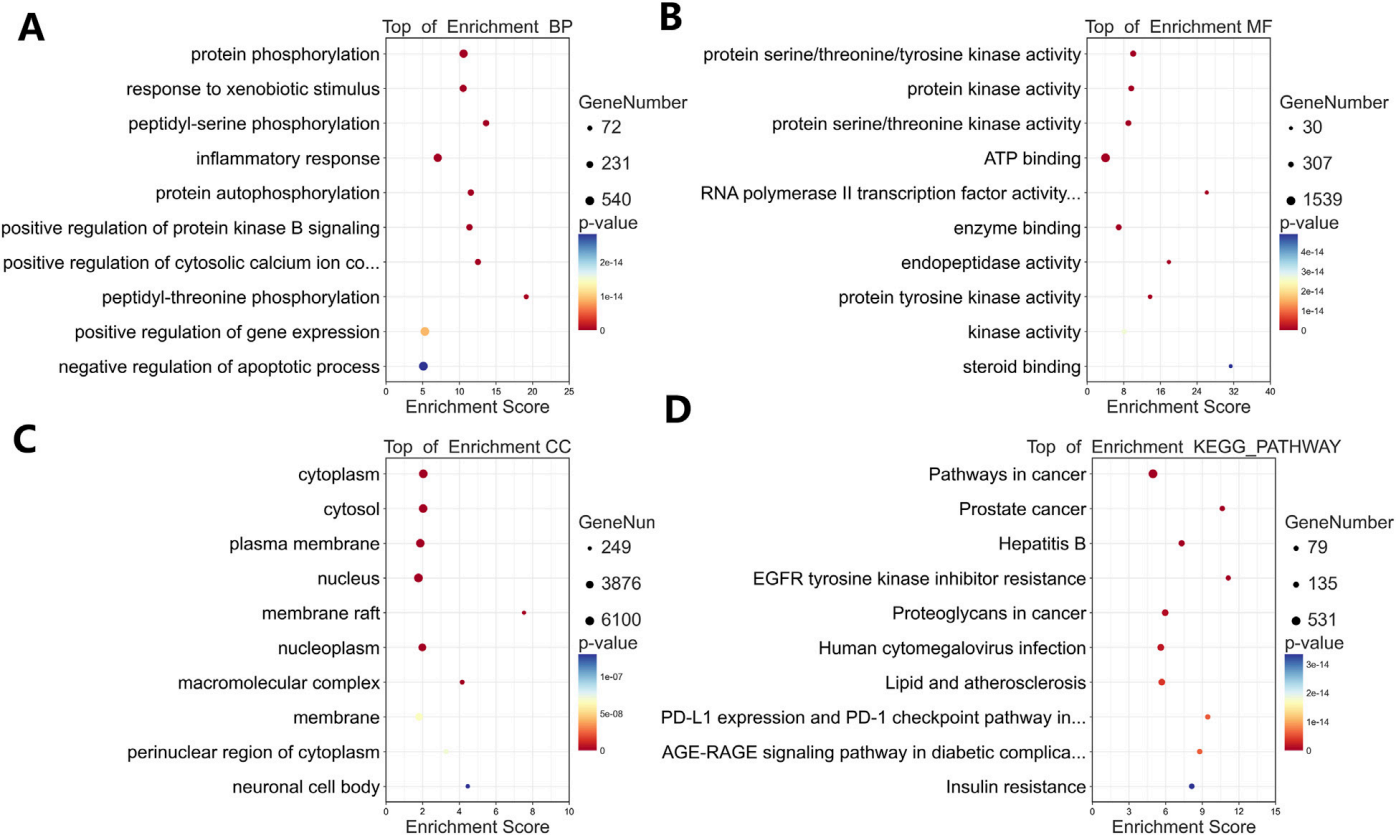
Functional enrichment analysis of TWP targets. Bubble plots show top 10 enriched terms for: **(A)** Biological processes (BP); **(B)** Molecular functions (MF); **(C)** Cellular components (CC); **(D)** KEGG pathways.

GO and KEGG analyses of the 242 targets identified 693 biological processes (BP), 95 cellular components (CC), 187 molecular functions (MF), and 167 KEGG pathways significantly enriched (p < 0.01). Top enriched terms (Figure 4) included: BP: Protein phosphorylation, response to xenobiotic stimuli, peptidyl-serine phosphorylation, inflammatory response (Figure 4A). MF: Protein kinase activity, ATP binding, enzyme binding, protein serine/threonine/tyrosine kinase activity (Figure 4B). CC: Cytoplasm, cytosol, plasma membrane, extracellular exosome (Figure 4C). KEGG: Cancer pathways (Pathways in cancer, Prostate cancer), signaling cascades (EGFR tyrosine kinase inhibitor resistance), and immune regulation (PD-L1/PD-1 checkpoint pathway) (Figure 4D). These results suggest TWP modulates proliferation, apoptosis, inflammation, and immune responses via multi-pathway regulation.

### 3.6 Microarray validation of core targets in clinical datasets

**FIGURE 5:**
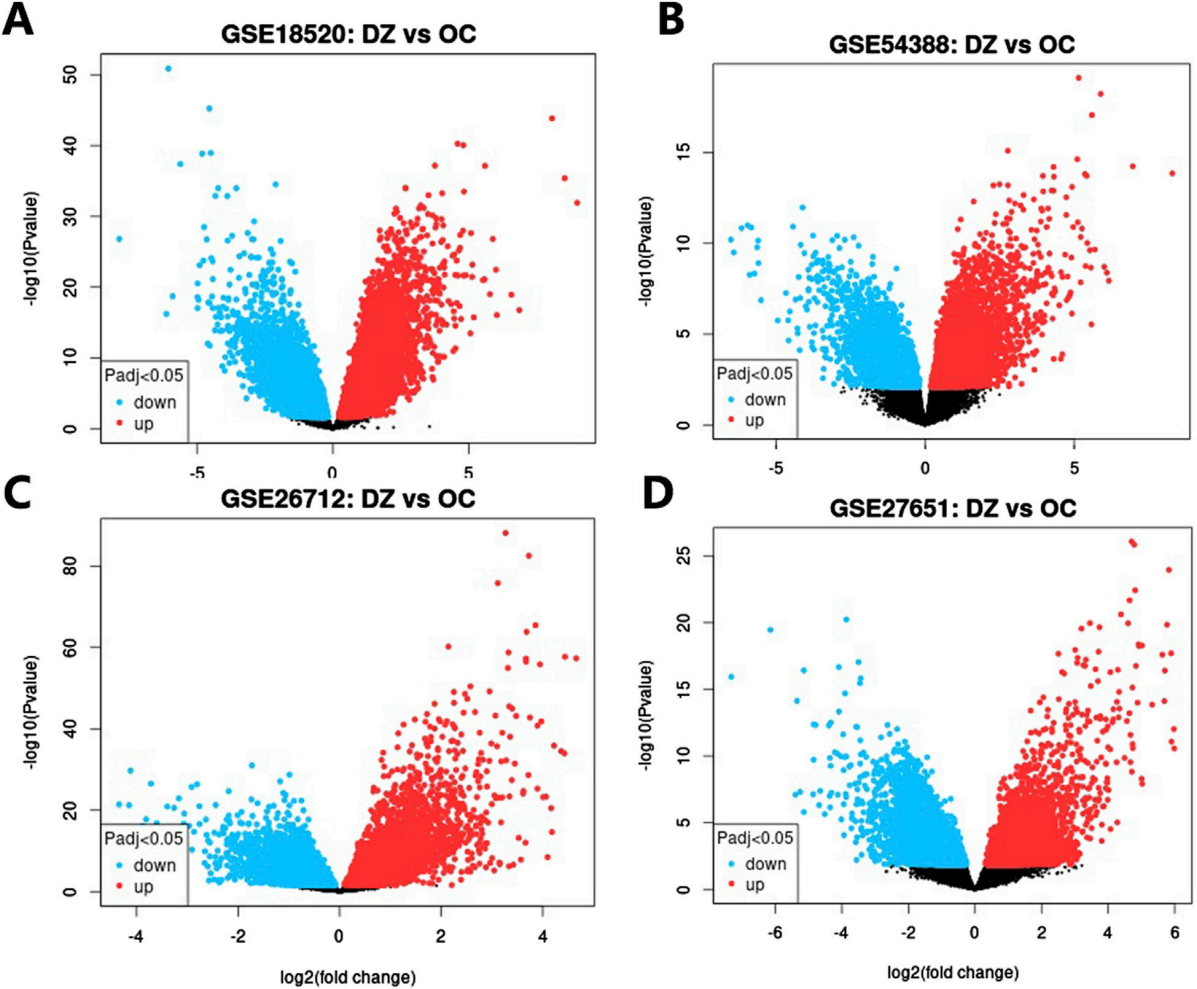
Volcano plots of DEGs in ovarian cancer datasets. **(A)** GSE18520, **(B)** GSE54388, **(C)** GSE26712, **(D)** GSE27651. Red dots: Upregulated genes (log_2_FC ≥ 1, adj. p < 0.05); Blue dots: Downregulated genes (log_2_FC ≤ −1, adj. p < 0.05); Gray dots: Non-significant genes.

Four independent ovarian cancer microarray datasets (GSE18520, GSE26712, GSE27651, GSE54388) were analyzed to validate the clinical relevance of hub genes. Differentially expressed genes (DEGs) were identified using stringent criteria (adjusted p < 0.05, |log2(FC)|≥1), and volcano plots were generated to visualize the distribution of gene expression changes (Figure 5). The analysis revealed substantial transcriptional alterations across datasets, with 723–3,254 upregulated and 1,296–3,568 downregulated genes per dataset. Among the top 10 hub genes, five core targets (PTPN11, JAK2, EGFR, SRD5A1, and JAK1) demonstrated consistent differential expression in ≥2 datasets (Table 4). These five genes, which demonstrated both network centrality and differential expression in clinical samples, were designated as core targets for subsequent molecular docking analysis. Their differential expression patterns in ovarian cancer tissues suggest potential roles in disease pathogenesis and progression, further supporting their relevance as therapeutic targets for TWP.

**TABLE 4:** Integrated analysis of GEO datasets and core target gene expression.

Dataset	Platform	Control	Affected	Downregulated	Stable	Upregulated	DEGs of core target genes
GSE54388	GPL570	6	16	1,877	2,884	1,266	PTPN11
GSE27651	GPL570	6	43	3,568	4,805	3,254	JAK2, EGFR, SRD5A1
GSE18520	GPL570	10	53	2,671	10,110	2,984	PTPN11, JAK2, SRD5A1, JAK1
GSE26712	GPL570	10	185	1,296	6,678	723	EGFR

### 3.7 Molecular docking validation of TWP active constituents against core targets

**FIGURE 6:**
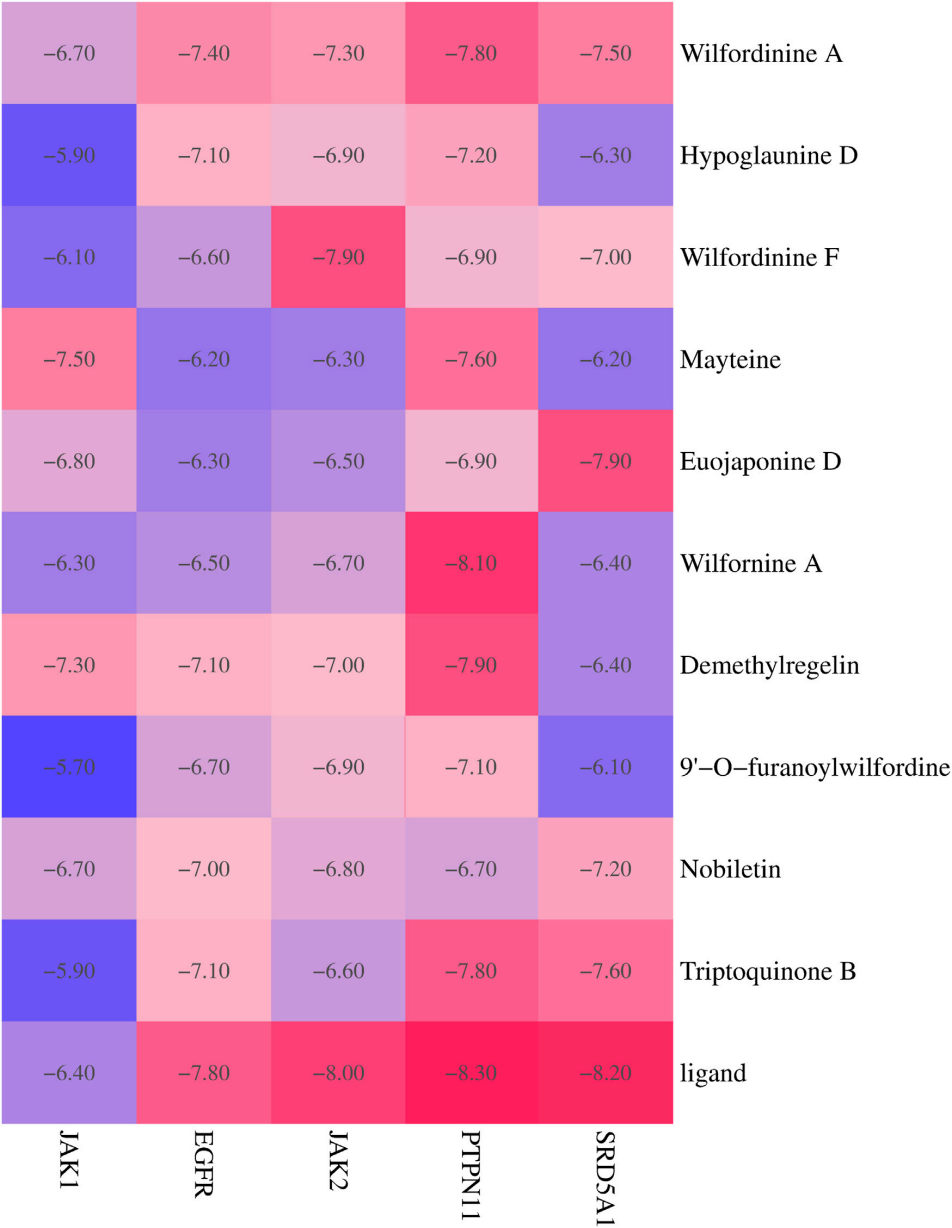
Heat map of docking energies (kcal/mol) between the top 10 TWP constituents and five core targets. Darker red indicates stronger binding.

**FIGURE 7:**
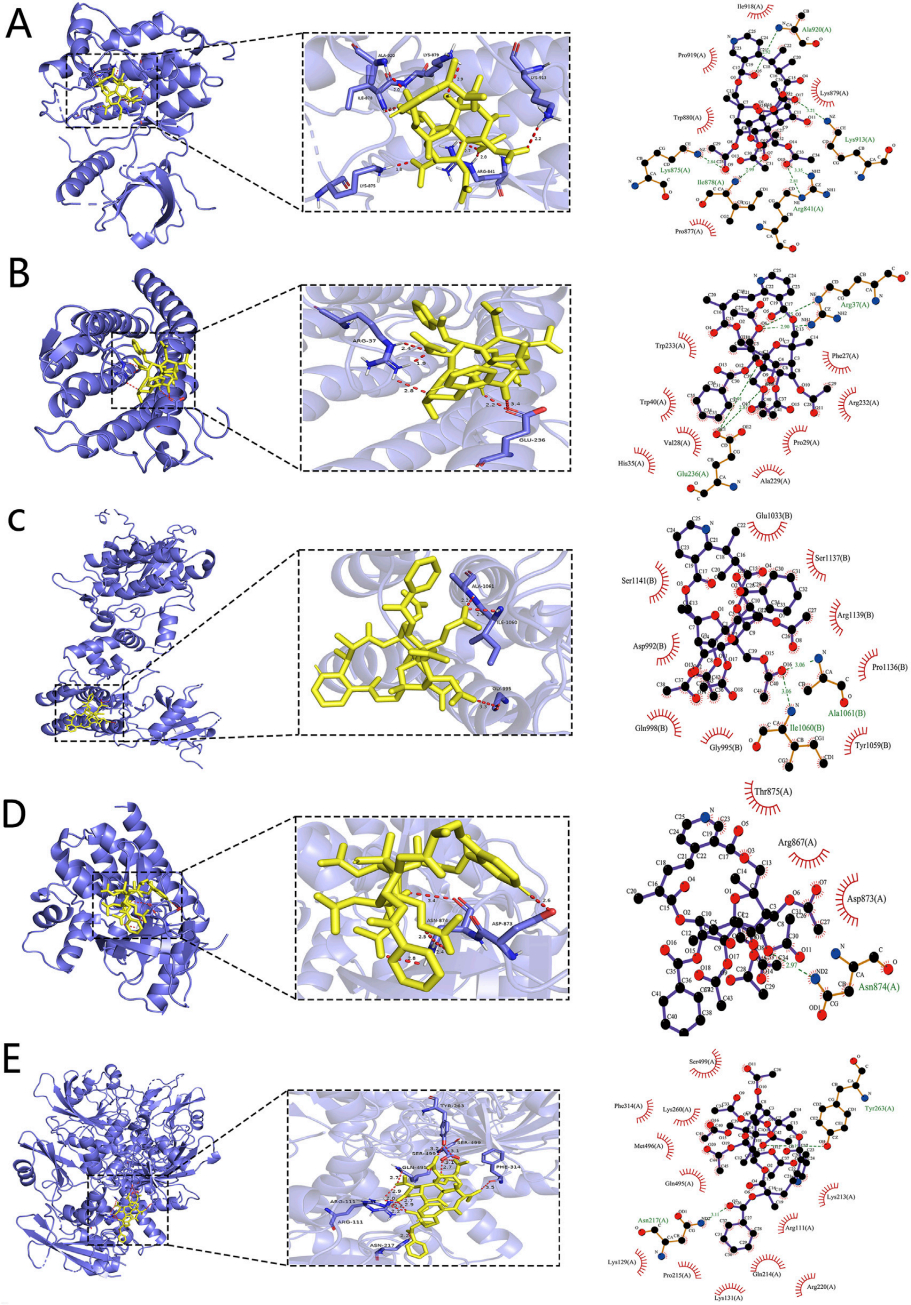
Representative binding poses of the optimal TWP ligands in the active sites of core targets. **(A)** EGFR-Wilfordinine A; **(B)** JAK1-Mayteine; **(C)** JAK2-Wilfordinine F; **(D)** PTPN11-Wilfornine A; **(E)** SRD5A1-Euojaponine D. Left panels: overall protein cartoon (blue) with ligand (yellow sticks) and binding-site box (dashed line). Middle panels: close-up 3D interactions (hydrogen bonds as dashed yellow lines, distances in Å). Right panels: 2D interaction diagrams.

To systematically evaluate the binding potential of representative TWP constituents toward the five core targets predicted by network pharmacology (EGFR, JAK1, JAK2, PTPN11, and SRD5A1), molecular docking was performed with AutoDock Vina for the ten highest-connected TWP compounds. Binding free energy (ΔG) ≤ −5 kcal/mol was set as the threshold for effective binding, and ΔG ≤ −7 kcal/mol was defined as strong binding (Ge et al., 2024). Heat-map analysis (Figure 6) revealed that all the compound–target pairs exhibited ΔG ≤ −5 kcal/mol, of which 27 pairs reached ΔG ≤ −7 kcal/mol, indicating broad and robust binding propensity. Comparative evaluation identified the optimal ligand for each target: EGFR-Wilfordinine A (−9.2 kcal/mol), JAK1-Mayteine (−8.8 kcal/mol), JAK2-Wilfordinine F (−8.5 kcal/mol), PTPN11-Wilfornine A (−7.8 kcal/mol), SRD5A1-Euojaponine D (−9.5 kcal/mol). Detailed pose analysis (Figure 7) showed that TWP constituents anchor within the catalytic pockets via hydrogen bonding, hydrophobic contacts, π-π stacking, and van der Waals forces. Specifically, Wilfordinine A forms key hydrogen bonds with EGFR Lys745 and Met793, whereas Euojaponine D establishes an extensive hydrophobic network within the SRD5A1 binding cavity. These findings corroborate the network-predicted targets and provide structural insights into the multitarget molecular basis underlying TWP efficacy in ovarian cancer therapy.

### 3.8 MD simulation analysis

**FIGURE 8:**
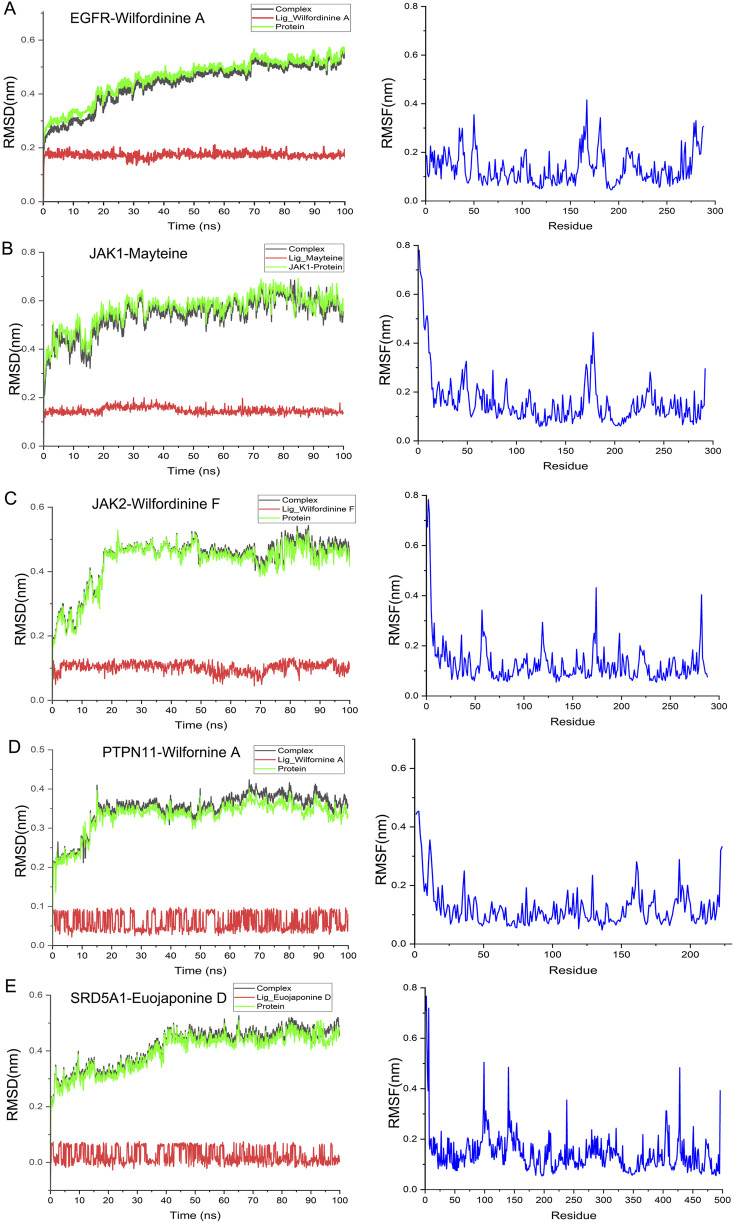
MD stability analyses of the five optimal TWP–target complexes. **(A)** EGFR-Wilfordinine A; **(B)** JAK1-Mayteine; **(C)** JAK2-Wilfordinine F; **(D)** PTPN11-Wilfornine A; **(E)** SRD5A1-Euojaponine D; Left panels: RMSD evolution of the complex (black), protein (green) and ligand (red). Right panels: per-residue RMSF (blue).

To probe the thermodynamic stability and conformational dynamics of the top-ranked complexes, 100-ns all-atom MD simulations were performed for the five optimal pairs identified in Section 3.7: EGFR-Wilfordinine A, JAK1-Mayteine, JAK2-Wilfordinine F, PTPN11-Wilfornine A, and SRD5A1-Euojaponine D. Structural stability and residue flexibility were quantified via RMSD and RMSF, respectively. RMSD trajectories (Figure 8, left) indicate rapid convergence to equilibrium for all systems. EGFR-Wilfordinine A and JAK1-Mayteine plateau at ∼0.5–0.6 nm after 15–20 ns, whereas JAK2-Wilfordinine F stabilizes similarly at ∼0.6 nm PTPN11-Wilfornine A exhibits the lowest RMSD (∼0.35 nm), reflecting a compact binding mode. SRD5A1-Euojaponine D undergoes a 30-ns relaxation phase before stabilizing at ∼0.45 nm, consistent with initial pocket remodeling. Ligand RMSDs remain <0.2 nm throughout, confirming persistent binding poses. RMSF profiles (Figure 8, right) reveal that flexible loops and termini account for most residue mobility, while catalytic cores retain rigidity. EGFR shows flexibility peaks at residues 50-75, 175-200 and 280-300; JAK1 and JAK2 exhibit elevated N-terminal and loop fluctuations; PTPN11 displays moderate, uniform mobility; SRD5A1 presents pronounced flexibility in residues 50-100 and 350-450. Collectively, the converged RMSD and restrained RMSF of binding-site residues corroborate the docking-derived poses and underscore the reliability of the predicted TWP-target interactions.

### 3.9 TWP suppresses ovarian cancer cell viability

**FIGURE 9:**
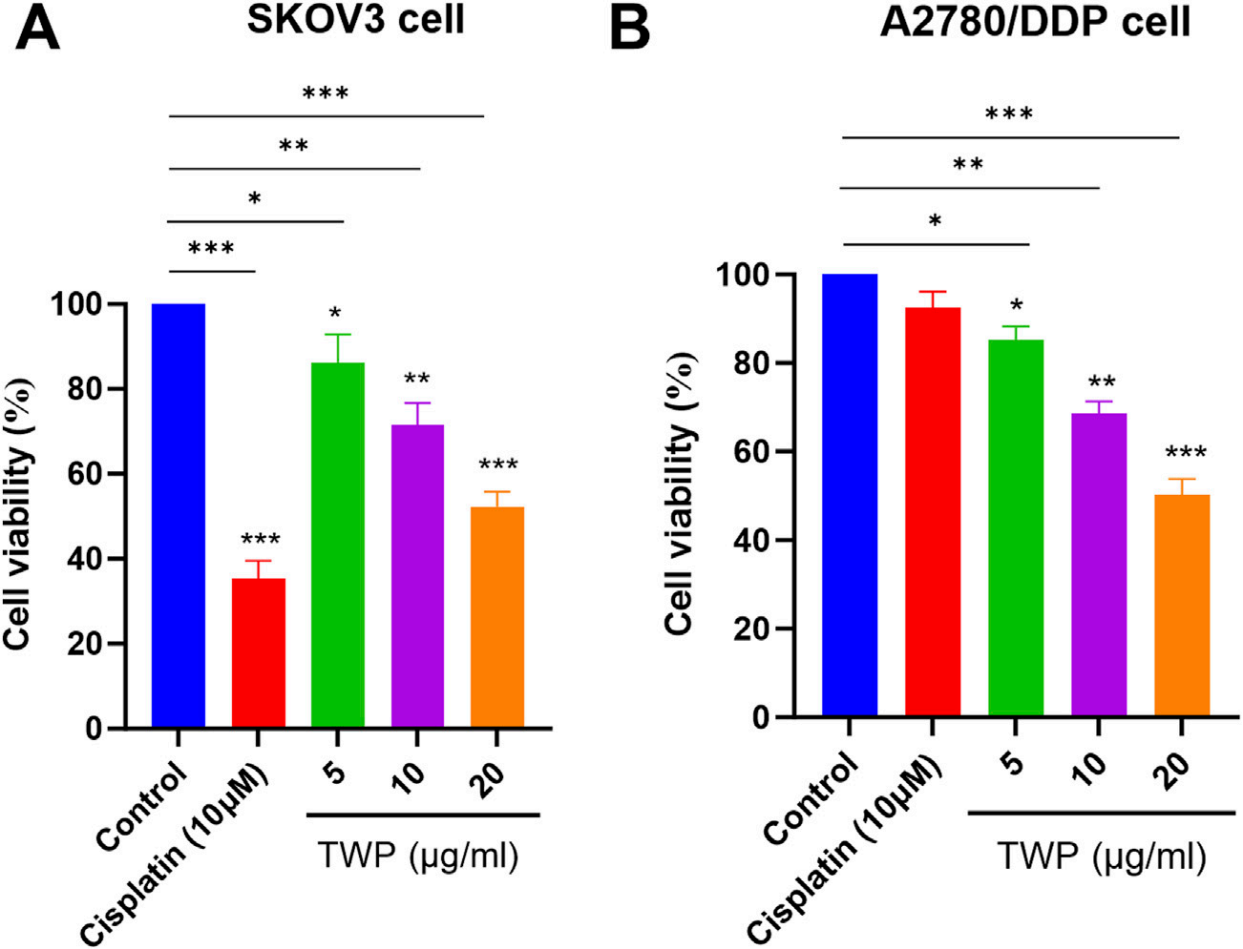
TWP inhibits ovarian cancer cell viability. **(A)** SKOV3 and **(B)** A2780/DDP cells were treated with TWP (5, 10, 20 μg/mL) or cisplatin (10 μM) for 24 h. Data are presented as mean ± SD. *P < 0.05, **P < 0.01,***P < 0.001 by ANOVA with Tukey *post hoc* test.

The predicted antitumor activity of TWP was first verified by CCK-8 assay in SKOV3 and cisplatin-resistant A2780/DDP cells. After 24 h exposure, TWP induced a concentration-dependent reduction in viability in both lines (Figure 9). In SKOV3 cells, 5, 10 and 20 μg/mL TWP decreased viability to 86.2% ± 3.1%, 71.5% ± 2.8% and 52.3% ± 3.5%, respectively. Comparable efficacy was observed in A2780/DDP cells (85.2% ± 3.1%, 68.5% ± 2.8% and 50.3% ± 3.5%). As expected, 10 μM cisplatin strongly inhibited SKOV3 cells (33.3% ± 2.4% viability) but exerted minimal cytotoxicity on A2780/DDP cells (92.5% ± 3.7%), confirming drug resistance. Notably, TWP retained potent activity against the resistant line, underscoring its therapeutic potential for cisplatin-refractory ovarian cancer.

### 3.10 TWP induces apoptosis in cisplatin-resistant cells

**FIGURE 10:**
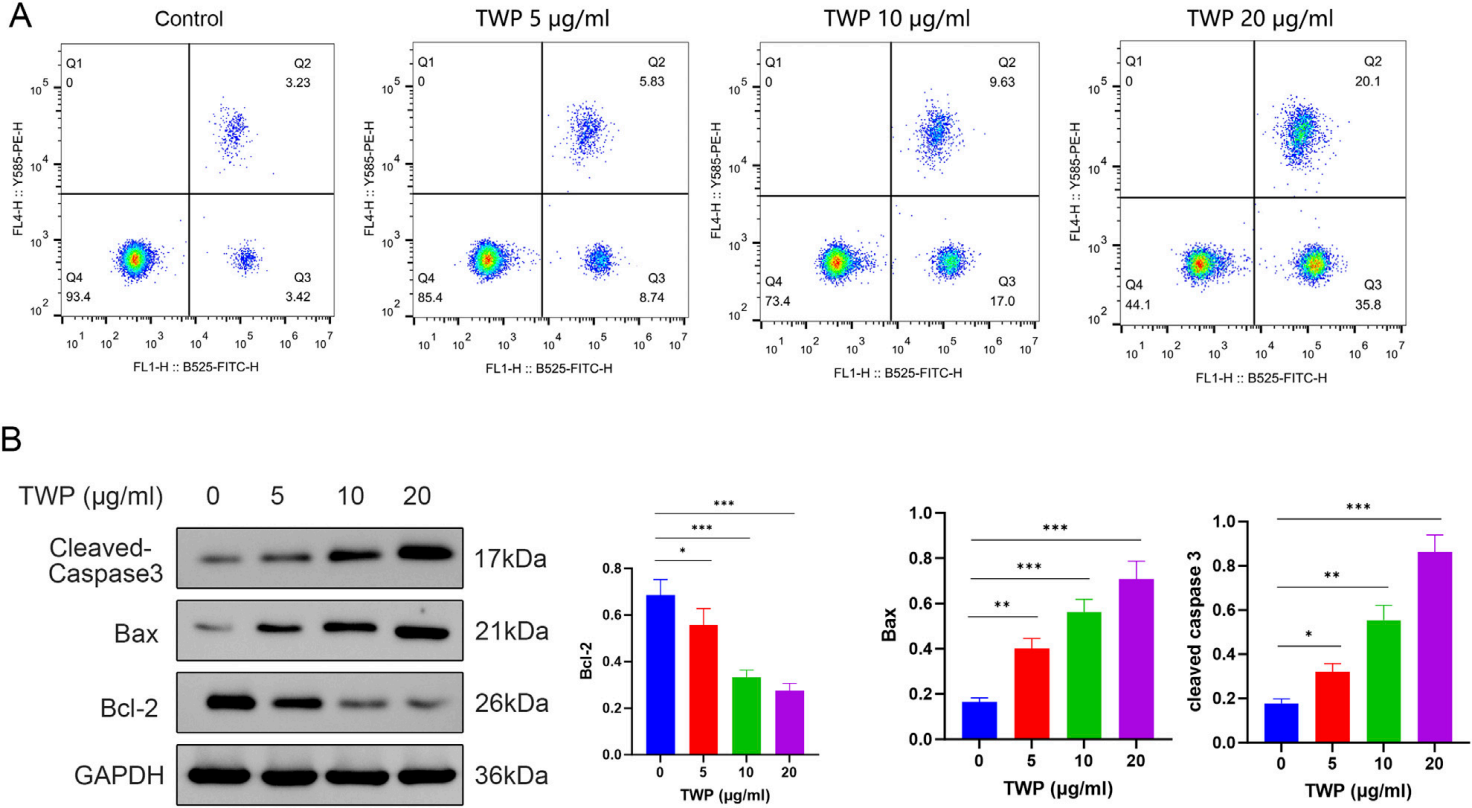
TWP dose-dependently induces apoptosis in A2780/DDP cells. **(A)** Representative dot plots and quantitative summary of Annexin V-FITC/PI staining; quadrants Q1-Q4 denote debris, late-apoptotic, early-apoptotic and viable cells, respectively. **(B)** Western blot and densitometry of Bcl-2, Bax and cleaved Caspase-3. GAPDH served as loading control. Data are presented as mean ± SD. *P < 0.05, **P < 0.01, ***P < 0.001 by ANOVA with Tukey *post hoc* test.

To determine whether TWP triggers programmed cell death in the cisplatin-resistant A2780/DDP line, Annexin V-FITC/PI flow cytometry and Western blot were performed. After 24 h exposure, TWP dose-dependently increased total apoptosis (early + late) from 6.65% (control) to 14.57%, 26.63% and 55.9% at 5, 10 and 20 μg/mL, respectively (Figure 10A). Western blot analysis revealed a corresponding molecular signature: Bax and cleaved Caspase-3 were upregulated, whereas Bcl-2 was downregulated, yielding a marked reduction in the Bcl-2/Bax ratio (Figure 10B). These data demonstrate that TWP overcomes cisplatin resistance by shifting the Bcl-2 family balance and activating the Caspase-3 mediated intrinsic apoptotic pathway.

### 3.11 TWP suppresses EGFR/JAK/PTPN11 signaling in cisplatin-resistant A2780/DDP cells

**FIGURE 11:**
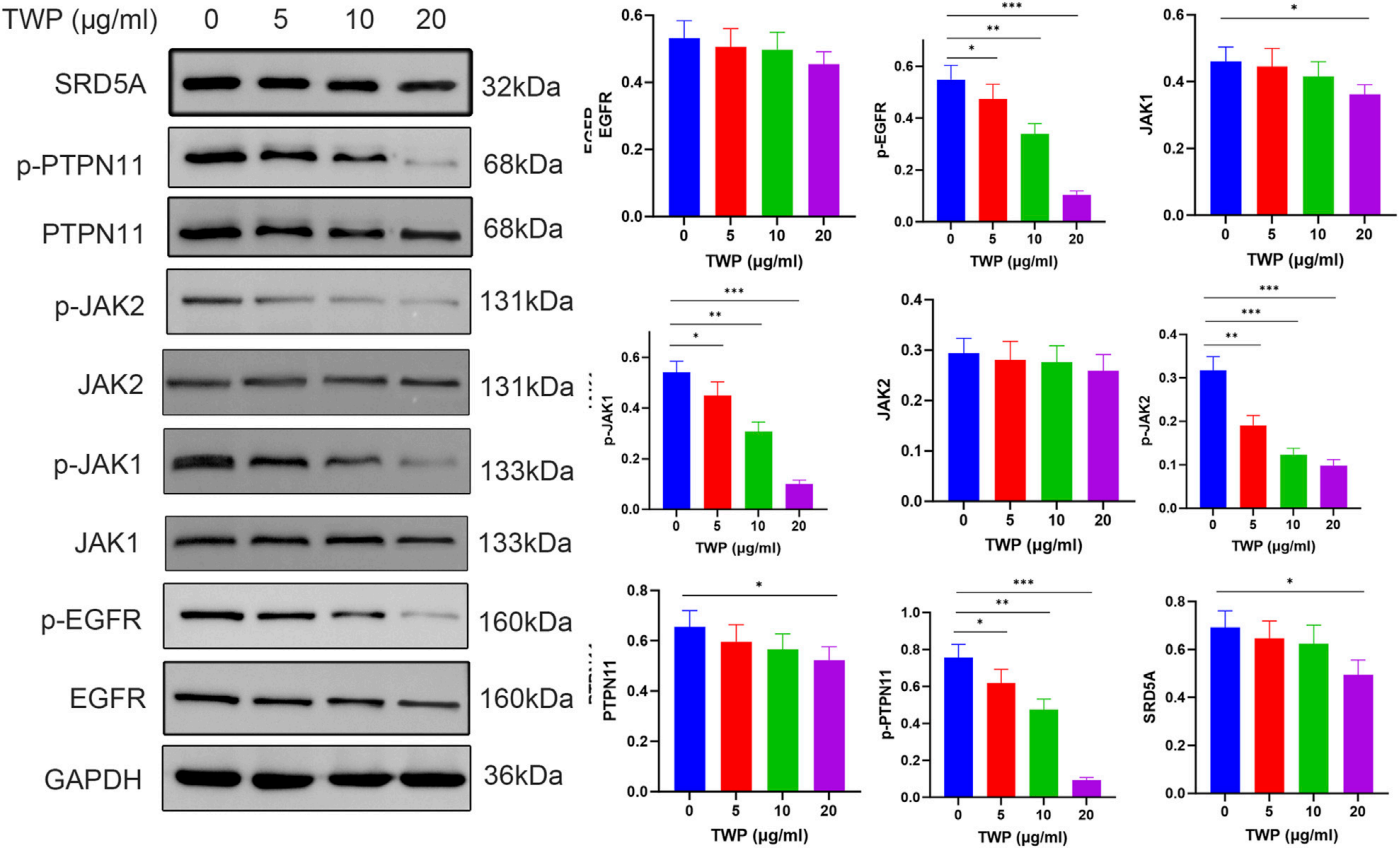
TWP inhibits EGFR/JAK/PTPN11 phosphorylation in A2780/DDP cells. (Left) Representative western blots. (Right) Quantification normalized to GAPDH. Data are presented as mean ± SD. *P < 0.05, **P < 0.01, ***P < 0.001 by ANOVA with Tukey *post hoc* test.

To verify the network-pharmacology predictions, A2780/DDP cells were treated with TWP (0–20 μg/mL) for 24 h and analyzed by Western blot analysis. As shown in Figure 11, TWP produced a concentration-dependent reduction in the phosphorylated forms of EGFR, JAK1, JAK2 and PTPN11; significant inhibition was evident at 5 μg/mL and maximal at 20 μg/mL (*P* < 0.001). Total EGFR and JAK2 levels remained unchanged, whereas total JAK1 and PTPN11 were modestly decreased only at 20 μg/mL (*P* < 0.05). SRD5A1 expression declined markedly at the same dose (*P* < 0.05). These results demonstrate that TWP primarily inactivates upstream kinases/phosphatases through de-phosphorylation, with secondary downregulation of select targets at higher concentrations, thereby disrupting multiple pro-growth circuits in cisplatin-resistant ovarian cancer cells.

### 3.12 TWP inhibits PI3K-AKT, JAK-STAT, and ERK-MAPK signaling

**FIGURE 12:**
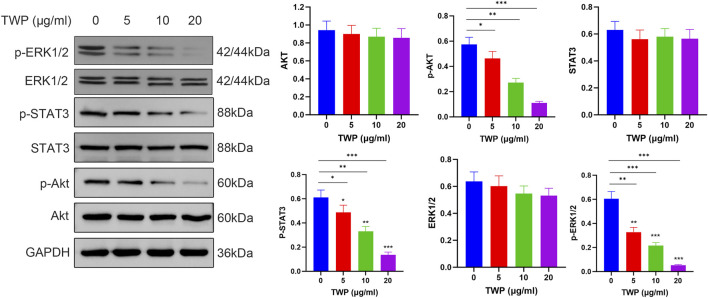
TWP inhibits phosphorylation of Akt, STAT3, and ERK1/2 in A2780/DDP cells. Left panel: Representative western blots showing the effects of TWP (0, 5, 10, 20 μg/mL) on the phosphorylation and total protein levels of Akt, STAT3, and ERK1/2 in A2780/DDP cells. Molecular weights (kDa) are indicated. Right panels: Quantitative analysis of protein levels normalized to GAPDH. Data are presented as mean ± SD. *P < 0.05, **P < 0.01, ***P < 0.001 by ANOVA with Tukey *post hoc* test.

To verify the downstream consequences of TWP-mediated inhibition of upstream signaling molecules described in section 2.11, we investigated the effects of TWP on three major signaling cascades: PI3K-AKT, JAK-STAT, and ERK-MAPK pathways. Western blot analysis (Figure 12) demonstrated that TWP treatment resulted in a concentration-dependent decrease in the phosphorylation levels of Akt, STAT3, and ERK1/2 in A2780/DDP cells. Importantly, total protein levels of Akt, STAT3 and ERK1/2 remained unaltered across the entire concentration range, confirming that TWP modulates pathway activity through post-translational de-phosphorylation rather than transcriptional or translational repression. These results provide direct experimental evidence that TWP simultaneously suppresses the activation of these three critical pro-survival signaling pathways in cisplatin-resistant ovarian cancer cells.

## 4 Discussion

Ovarian cancer resistance to platinum-based chemotherapy represents a multifactorial process involving dysregulated DNA repair, altered drug transport, and aberrant survival signaling (Xiong et al., 2024). Conventional single-target agents often fail due to tumor heterogeneity and compensatory pathway activation, highlighting the need for multi-target strategies (Doostmohammadi et al., 2024). Our integrated approach, bridging UPLC/Q-TOF-MS analysis with computational prediction and experimental validation, has elucidated a coherent mechanistic framework for TWP’s ability to overcome this resistance.

We identified that alkaloids represent a major bioactive class within TWP and demonstrated that they execute a multi-pronged attack on a hub of signaling proteins crucial for chemoresistance. This finding expands the current understanding of Tripterygium’s therapeutic potential, suggesting that alkaloids—alongside diterpenoids—may contribute significantly to its anti-tumor activity through complementary mechanisms. Emerging evidence indicates that alkaloids exhibit distinct pharmacokinetic advantages, including enhanced bioavailability and multi-target modulation of apoptosis, oxidative stress, and chemosensitization pathways (Mondal et al., 2019; Olofinsan et al., 2023).

Our investigation revealed that TWP’s efficacy is driven by its ability to modulate five core targets—EGFR, JAK1, JAK2, PTPN11, and SRD5A1—which our analysis showed to be dysregulated in clinical ovarian cancer datasets. The EGFR-PI3K-AKT and JAK-STAT pathways are frequently co-activated in platinum-resistant ovarian cancer, creating redundant survival signals that limit single-agent efficacy (Zhao et al., 2021). Simultaneously targeting these parallel pathways has emerged as a rational strategy to overcome therapeutic escape (Wen et al., 2015). The identification of PTPN11 and SRD5A1 as additional key targets represents a significant advance. PTPN11 (SHP2), a critical node in receptor tyrosine kinase (RTK) signaling, is consistently upregulated in ovarian cancer and promotes tumor progression via the PI3K-AKT and ERK-MAPK pathways (Fedele et al., 2018; Cao et al., 2022). Our data showing TWP-mediated PTPN11 inhibition suggests a mechanism to disrupt these oncogenic cascades, potentially circumventing resistance. Similarly, TWP’s suppression of SRD5A1 may attenuate androgen signaling, which contributes to progression in a significant subset of ovarian cancers, offering a novel strategy to counteract hormone-mediated chemoresistance (Mizushima and Miyamoto, 2019; Chung et al., 2021).

The consequence of this upstream, multi-target engagement is a profound and coordinated collapse of downstream signaling pathways. The observed blockade of Akt, STAT3, and ERK phosphorylation is not merely a downstream effect but the functional nexus of TWP’s anti-cancer action. By curtailing Akt activation, TWP deprives tumor cells of a critical pro-survival hub, re-sensitizing them to cisplatin-induced apoptosis (Li et al., 2024). Similarly, TWP-mediated inhibition of STAT3 phosphorylation dismantles a key driver of immune evasion, metastasis, and cancer stemness (Liu et al., 2025). Finally, the suppression of ERK phosphorylation interrupts the canonical MAPK cascade, blunting mitogenic signals (Lucas et al., 2022). This multi-node suppression generates a synergistic attenuation of the transcriptional programs governing proliferation and survival, which is the cornerstone of overcoming chemoresistance.

**FIGURE 13:**
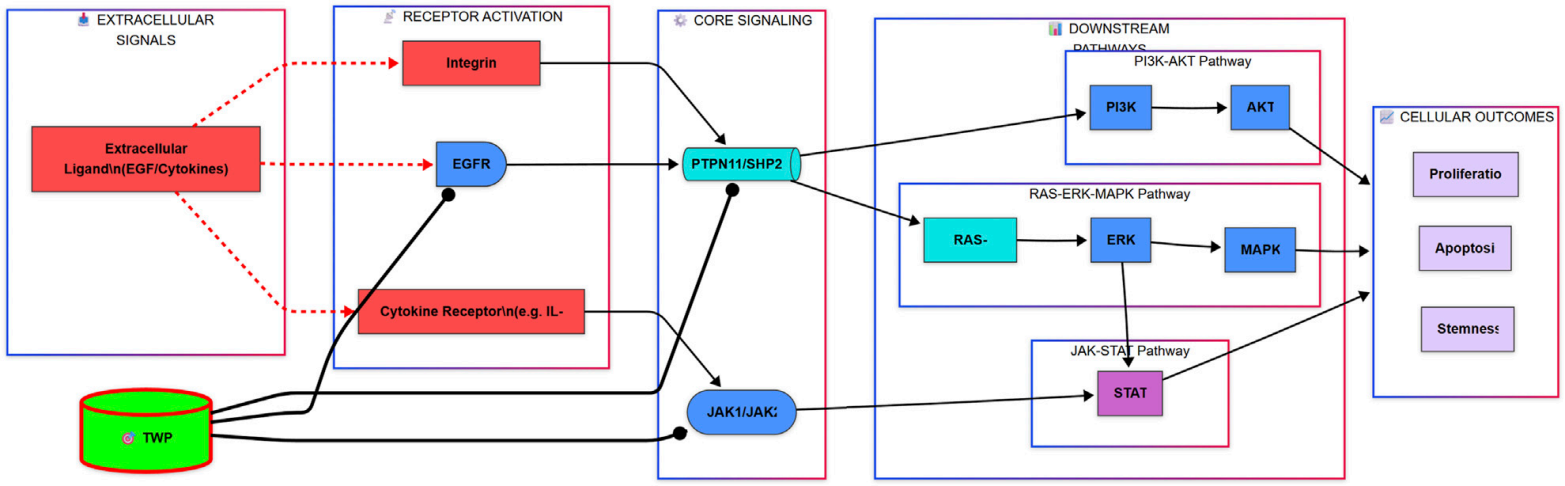
Proposed Mechanism for TWP-Mediated Reversal of Cisplatin Resistance in Ovarian Cancer. The alkaloid components of TWP act as multi-target inhibitors, engaging upstream regulators including EGFR, Cytokine Receptors (via JAK1/JAK2), and the critical signal integrator PTPN11/SHP2. This coordinated inhibition blocks signal transduction through three major oncogenic pathways: the JAK-STAT, RAS-ERK-MAPK, and PI3K-AKT cascades. Consequently, this leads to the suppression of pro-survival outputs such as proliferation and stemness, while promoting apoptosis, ultimately restoring cellular sensitivity to cisplatin.

As illustrated in the comprehensive signaling diagram (Figure 13), our findings paint a cohesive picture. TWP alkaloids act as inhibitors at the receptor and signal integration level, targeting EGFR, JAK1/2, and the pivotal phosphatase PTPN11/SHP2. This action blocks signals emanating from growth factors (e.g., EGF) and cytokines (e.g., IL-6), leading to the simultaneous shutdown of three distinct but interconnected oncogenic cascades: the JAK-STAT pathway, the Ras-ERK-MAPK pathway, and the PI3K-AKT pathway. The ultimate result is the inhibition of key malignant phenotypes, including proliferation and stemness, and the induction of apoptosis, thereby restoring cisplatin sensitivity.

The clinical implications of these findings are substantial, especially given the suboptimal outcomes in platinum-resistant ovarian cancer (PROC) (Eskander et al., 2023; Leung and Konstantinopoulos, 2021; Naumann and Coleman, 2011) The ability of TWP to simultaneously inhibit EGFR, JAK-STAT, PTPN11, and androgen signaling pathways suggests strong potential for synergy with existing targeted therapies. For instance, TWP-mediated PTPN11/SHP2 inhibition may prevent resistance to EGFR inhibitors (e.g., osimertinib) by blocking RAS-ERK-MAPK reactivation (Chen et al., 2024; Scheiter et al., 2024). Its suppression of PI3K-AKT signaling may synergize with PARP inhibitors by downregulating BRCA expression and exacerbating DNA repair deficiency, a strategy shown to be effective in platinum-resistant models (Bhamidipati et al., 2023; Xu et al., 2022).

The alkaloids we identified belong to a class of natural products that have been a cornerstone of cancer chemotherapy for decades, including vinca alkaloids and camptothecin derivatives (Olofinsan et al., 2023; Mondal et al., 2019). The strong binding affinities and stable interactions observed in our molecular dynamics simulations provide a structural blueprint for potentially optimizing these natural compounds into multi-target inhibitors with improved pharmacokinetic profiles, following the successful trajectory of many other plant-derived anti-cancer agents.

Crucially, TWP is already an approved prescription drug in China for inflammatory and autoimmune indications. This existing clinical foundation confers notable translational advantages over *de novo* chemical entities: established manufacturing and quality control, known safety management, and clinical accessibility. Mechanistic elucidation of its anti-tumor actions provides a rational basis for indication expansion via drug repurposing, potentially accelerating its path into oncology, where platinum-resistant ovarian cancer represents a clear unmet need. Such a “mechanism-to-medicine” trajectory—grounded in multi-target network disruption—supports pragmatic clinical exploration, including rational combinations with targeted or DNA damage response agents.

## 5 Limitations

Despite these advances, several limitations warrant acknowledgment. First, our validation was restricted to *in vitro* models; future studies must employ patient-derived xenografts (PDX) or organoids to recapitulate the complex tumor microenvironment (Yoshida, 2020). Second, our *in vitro* validation was conducted using the total TWP extract rather than an alkaloid-enriched fraction or isolated compounds. This approach was chosen for its direct clinical relevance, as TWP is the approved pharmaceutical preparation. However, this experimental design means that while our integrated *in silico* and experimental evidence strongly points to alkaloids as key mediators, we cannot definitively attribute the observed anti-tumor effects solely to this chemical class. Consequently, the precise relative contributions of individual compounds and their potential synergies—both among alkaloids and between alkaloids and other constituents like diterpenoids—remain unresolved (Wu et al., 2022). Future work with isolated active alkaloids is necessary to deconstruct these complex interactions. Third, the heterogeneity of ovarian cancer subtypes was not addressed, and validation in non-serous carcinomas is an essential next step.

## 6 Conclusion

This study proposes a mechanism potentially centered on alkaloid constituents by which TWP may help overcomes cisplatin resistance in ovarian cancer. Integrated computational analyses and *in vitro* assays suggest that TWP engages and inactivates EGFR, JAK1, JAK2, and PTPN11, while downregulating SRD5A1, thereby coordinating suppression of PI3K-AKT, JAK-STAT, and ERK-MAPK signaling. The resulting disruption of redundant survival circuits is associated with apoptosis induction and re-sensitization of resistant cells. While non-alkaloid constituents may also contribute to these effects, the findings provide strong, albeit indirect, support for an alkaloid-driven multi-target synergy. These mechanistic insights not only rationalize the observed anti-tumor activity but also support the potential clinical repurposing of an approved medicine toward oncology indications, and highlight the need for future validation using alkaloid-enriched preparations or isolated monomers.

## Data Availability

The original contributions presented in the study are included in the article/Supplementary Material, further inquiries can be directed to the corresponding author; GEO datasets used are GSE18520, GSE54388, GSE26712, GSE27651.
